# Gene function prediction based on genomic context clustering and discriminative learning: an application to bacteriophages

**DOI:** 10.1186/1471-2105-8-S4-S6

**Published:** 2007-05-22

**Authors:** Jason Li, Saman K Halgamuge, Christopher I Kells, Sen-Lin Tang

**Affiliations:** 1 Dynamic Systems & Control Group, DoMME, University of Melbourne, Melbourne, Australia; 2 Research Center for Biodiversity, Academia Sinica, Taipei, Taiwan

## Abstract

**Background:**

Existing methods for whole-genome comparisons require prior knowledge of related species and provide little automation in the function prediction process. Bacteriophage genomes are an example that cannot be easily analyzed by these methods. This work addresses these shortcomings and aims to provide an automated prediction system of gene function.

**Results:**

We have developed a novel system called SynFPS to perform gene function prediction over completed genomes. The prediction system is initialized by clustering a large collection of weakly related genomes into groups based on their resemblance in gene distribution. From each individual group, data are then extracted and used to train a Support Vector Machine that makes gene function predictions. Experiments were conducted with 9 different gene functions over 296 bacteriophage genomes. Cross validation results gave an average prediction accuracy of ~80%, which is comparable to other genomic-context based prediction methods. Functional predictions are also made on 3 uncharacterized genes and 12 genes that cannot be identified by sequence alignment. The software is publicly available at .

**Conclusion:**

The proposed system employs genomic context to predict gene function and detect gene correspondence in whole-genome comparisons. Although our experimental focus is on bacteriophages, the method may be extended to other microbial genomes as they share a number of similar characteristics with phage genomes such as gene order conservation.

## Background

The increasing number of completely sequenced genomes has enabled gene function predictions by means of whole genome comparison. Existing methods such as SynBrowse [1], Vista [2], LAGAN [3], PipMaker [4] and Ensembl SyntenyView [5] provide visualization of conserved regions between two or more genome sequences for comparative analysis. Such visualization facilitates the prediction of gene function based on comparison of genomic context information such as co-occurrence of genes [6,7] and conservation of gene order [8,9].

However, these methods have two major limitations. First, they rely on sequence alignment to identify corresponding genes or regions between genomes [1-5,10-12]. Consequently, they cannot automatically detect homologous or functionally similar genes that share no sequence similarity, resulting in a need for manual prediction for those genes. Second, these methods require the genomes being compared to be closely related. This hinders the possibility of automatically analyzing a large collection of weakly related genomes and makes it impossible to inspect a genome to which related species have not been identified.

Bacteriophage genomes are one example that suffers from the above limitations. Firstly, sequence alignment based methods are not fully reliable in detecting functionally similar genes within phages. This is because homologous phage genes have often diverged beyond the recognition of sequence similarity [13-15]. A key argument to explain such divergence was that the genes have a very distant common ancestry [15]. Secondly, requiring to compare only a few related phages and to ignore the remainder can hinder the genomic analysis of the target phage. The reason is that the global phage relationships are not clearly defined phylogenetically due to an extensive amount of horizontal gene transfers (HGT) [14,16], implying that relatedness between phages often cannot be established. Consequently, it is desirable to have an objective measure to automatically identify closely related genomes based on the genetic data, as opposed to depending on the user to define a set of "related species".

This work addresses the shortcomings of the existing methods and aims to provide a highly automated gene function prediction system based on whole-genome comparison. The system, named SynFPS, contains two automated learning units with distinct roles: a clustering technique that utilizes gene-to-gene distances to identify closely related genomes and a Support Vector Machine (SVM) for discriminative classification on gene functions. The algorithm of SynFPS and the results of function prediction on phage genes will be presented in the remainder of this paper.

## Results and discussion

### Evaluation of prediction results by leave-one-out cross validation

We have attempted to perform predictions over nine common phage genes using SynFPS. These are major head, major tail, tape measure, prohead protease, integrase, terminase, portal, holin and lysin genes. They were selected on the basis of regular existence – they encode necessary functions not provided by their hosts, including structural and assembly genes, as well as lysis genes [16]. These genes were searched against the annotation database using regular expression patterns defined in Table 1. Manual modifications of the search results have been conducted to remove ambiguous entries.

Table 2 indicates the amount of genes that can be detected if sequence alignment (BLAST) alone was used. The K-Means clustering result based on these genes can be found in Supplementary Material (see Additional file 1).

We perform leave-one-out (LOO) cross validation to evaluate the prediction performances for these genes. For each gene function, we run the cross validation in each cluster individually over a discrete range of values of the kernel parameter – *σ *for Gaussian RBF kernel [17]. The *σ *value that gives the best accuracy is chosen and is used for all future predictions for that function. The prediction accuracies shown in Table 3 are the averages of cross validation results across all the clusters.

*K*-fold cross validation may also be used to evaluate the prediction performances and it is expected that accuracies are lower with a smaller *K *value. For instance, the prediction accuracy for Terminase is 79.8% for *K *= 4 and 62.3% for *K *= 2. However, LOO is more suited to our overall purpose – one primary objective of the cross validation is to find out the near optimal *σ *value for the gene class to perform future predictions. Since most clusters contain only a very small portion of genomes that require genuine prediction, they are best simulated by LOO, where only one genome is taken out for prediction testing at a time.

The prediction accuracies are averaged at ~80%. The 100% prediction accuracy of lysin can be explained by the strong context relationship between lysin and holin. Since the presence of a lysin is always accompanied by the presence of a holin immediately beside it [18], SynFPS can easily identify the lysin gene if it already knows the position of the holin. However, the converse is not true: the identification of holin genes may not depend upon the presence of lysin. Consequently, the prediction accuracy for holin is not as high.

These prediction accuracies reflect the sensitivity of the system (true positives/(true positives + false negatives)). The specificity of the system (true negatives/(true negatives + false positives)) on the other hand is always larger the sensitivity because of two system features. Firstly, we allow only a single positive prediction for each genome (see Methods). Thus, the number of false negatives is always the same as the number of false positives, implying that the specificities always scale together with the sensitivities. Secondly, the number of negative training data (hence true negatives) is always larger than the number of positive training data (hence true positives), and consequently Specificity > Sensitivity. One reason for using LOO cross validation accuracies to evaluate the system is the lack of benchmark for our problem. However, it may be noteworthy that other genomic-context based methods for the prediction of functional elements have similar reported accuracies ranging from 72% to 80% [6].

### Trade-off between prediction coverage and prediction accuracy

We have examined the effect of the K-Means adaptive threshold *t *on the prediction accuracies. The value of *t *∈ (0,1] implicitly specifies the maximum tolerable distance between any two genomes within a cluster. As a result, as *t *→ 0, there are as many clusters as the number of genomes, and as *t *→ 1, there is only one cluster. Both of these cases do not provide useful information for prediction. Since there is no analytical method to find out a good value for *t*, we have run SynFPS over a range of values from *t *= 0.05 to *t *= 0.3. Values outside this range generate either too many or too few clusters (average number of genomes per cluster < 2 or number of clusters < 3 respectively). Using different *t *values lead to a different amount of genomes that are covered by the automated prediction (a.k.a. prediction coverage). Genomes within the "coverage" are those for which SynFPS has made a classification decision; the remaining genomes are discarded or ignored by SynFPS. Here are examples of genomes not in coverage:

• genomes not containing the gene being predicted (discarded during cross validation only)

• genomes that is in a cluster on their own

• within a cluster, if there are fewer than two genomes that contain the gene being predicted, then all the genomes are discarded

• genomes with genomic context different to the consensus of the group may be discarded

Figure 3 shows the plot of prediction accuracies versus prediction coverage. The highest coverage values for all gene functions are about 20–25%, achieved by using a *t *value ~0.1. The results indicate that we can obtain a higher accuracy by lowering the coverage. However, the ultimate purpose of the system is to make genuine predictions over the genomes that lack identification of the genes being predicted. Lowering the coverage can lead to ignorance of many of these genomes. Consequently, one must find a balance between the accuracy and the coverage according to the intended task.

### Functions predicted to 3 uncharacterised genes and 12 sequence dissimilar genes

Using the maximum coverage and the *σ *values optimized by LOO cross validation, we have generated predictions over genomes within which certain gene functions were not already detected. The outcome of SynFPS is to identify which genes within those genomes correspond to the functions of our interest. The prediction outcomes are listed in Table 4.

Three genes that we have predicted functions for have no existing functional annotation in the database (marked *uncharacterised *in Table 4). Seven genes in Table 4 exhibit sequence similarity to their reference genes, suggesting that their predicted functions are supported by both sequence similarity and the genomic context information embedded in our system, such as gene order conservation and positional coupling. For other genes that show no sequence similarity (a total of 12 of them in Table 4), the predicted functions are only evident by the genomic context. It is noteworthy that sequence alignment based methods would have failed in finding correspondences to these genes. Other prediction results have complemented existing annotations in the database in cases where they do exist, and therefore support the validity of our approach.

## Conclusion

We presented a novel genomic-context based method capable of predicting gene functions from a large collection of genomes. An adaptive K-Means clustering is used to distinguish groups of related genomes based on the conservation of gene order and the conservation of gene-to-gene distances. The clustering results serve as a platform for the SVM to extract training data to perform classification based predictions. Nine common gene functions of bacteriophages were tested and the LOO cross-validated prediction results are averaged at 80%. Functional predictions are also made on 3 uncharacterized genes and 12 genes that cannot be identified by sequence alignment.

Although our experimental focus is on bacteriophages, the method may be extended to other microbial genomes. For example, bacterial genomes have been observed with conserved gene order [8,19,20] and conserved gene-to-gene distances (positional coupling) [21,22]. These properties satisfy the underlying assumptions of our approach and suggest potential application of the method.

## Methods

### Strategy overview – SynFPS

**Figure 1:**
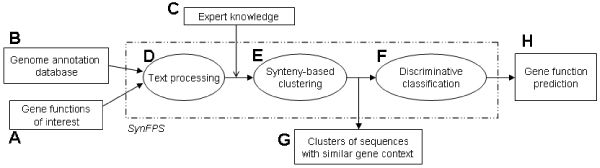
**Structure of the Synteny-based Function Prediction System (SynFPS)**. The dotted line represents the system boundary, outside of which lies the system inputs and outputs. A set of gene functions (A) specified in the form of regular expressions are matched against the genome database (B) via the text processing unit (D), which result may then be refined (C). A clustering system (E) based on the synteny scores of the matching genes brings together genomes that show conservation of gene order and position (G). Such information is used to generate a set of positive and negative data (genes) to train the classification system (F) that produces function prediction results (H).

We present a novel method called Synteny-based Function Prediction System (SynFPS) capable of predicting gene functions among completed genomes based on the conservation of gene order (synteny) and the conservation of gene-to-gene distance. An overview of SynFPS is shown in Figure 1. The genome annotation database as shown in the figure defines the scope of analysis for the system. In our work, it consists of 296 phage genomes retrieved from GenBank (see Additional file 1).

SynFPS runs on Windows and is publicly available. It was developed in C# and requires the free Microsoft .NET Framework 2.0 to run. Bioperl 1.4 [23] is needed for data retrieval from public databases. Workstations with a single CPU of ~3.0 GHz and 1 GB of RAM are sufficient for reasonable performance over a collection of ~300 phages.

### Identification of functionally similar genes using regular expression

The system begins by identifying in the database a collection of genes that correspond to a set of user-specified gene functions. Instead of using sequence similarity as in many other methods [1,2,4,5,12], SynFPS identifies functionally similar genes using regular expressions [24]. For example, to search for genes that encode the major head proteins of phages, one possible regular expression pattern is "(?<!minor)\b(head|capsid) protein". With this pattern, we are including genes that have been annotated with "head protein" or "capsid protein" except those with the prefix term "minor". The use of regular expression is aimed at tackling annotation discrepancies among coding sequences in databases that do not have vocabulary control. The regular expression syntax used in SynFPS follows the syntax defined for the .NET Framework [25].

Once a regular expression pattern is given, the system searches against the annotation data of all the genomes that have been supplied to the program. By default, it will identify coding sequence (CDS) regions in each of the genome and then try to match the patterns against their annotated features such as "product", "function" and "note". The set of annotated features that the search will perform over is customisable by the users. The search results can be visually displayed, where the genomes and matching genes are illustrated. The display is interactive in which annotations can be viewed and search results can be modified via manual addition and removal of genes.

Although genome annotation processes are often assisted by sequence alignment, many annotations are prepared manually by biologists who conducted research on the genomes. Therefore, the set of sequences found by annotation search could embrace functionally similar genes that show no sequence similarity. In the results section, we provide an assessment on sequence alignment in relation to regular expression search.

### K-Means clustering to identify similar genomic context

The annotation search process leads to a mapping of genes across the genomes. This mapping provides the necessary information for a context based clustering. Let *G *= {*g*_1_, *g*_2_,..., *g*_*n*_} be the set of all gene functions where *g *is a symbol representing a function and *n *is the total number of functional classes identified. Let *m *be the number of genomes in the database. We define *X*_*k *_⊆ *G*, *k *= 1,2,..., *m *to be the set of genes detected in genome *k *and *C*_*kl *_= *C*(*X*_*k*_, *X*_*l*_) = *X*_*k *_∩ *X*_*l *_to be the common set of genes between genomes *k *and *l*. The genomic-context distance between two genomes *k *and *l *is defined as:

Dkl=∑gi,gj∈Ckl;i<j[dk(gi,gj)−dl(gi,gj)]|Ckl|+p(|Xk∪Xl|−|Ckl|)     (1)
 MathType@MTEF@5@5@+=feaafiart1ev1aaatCvAUfKttLearuWrP9MDH5MBPbIqV92AaeXatLxBI9gBamXvP5wqSXMqHnxAJn0BKvguHDwzZbqegyvzYrwyUfgarqqtubsr4rNCHbGeaGqiA8vkIkVAFgIELiFeLkFeLk=iY=Hhbbf9v8qqaqFr0xc9pk0xbba9q8WqFfeaY=biLkVcLq=JHqVepeea0=as0db9vqpepesP0xe9Fve9Fve9GapdbaqaaeGacaGaaiaabeqaamqadiabaaGcbaGaemiraq0aaSbaaSqaaiabdUgaRjabdYgaSbqabaGccqGH9aqpdaWcaaqaamaaqafabaWaamWaaeaacqWGKbazdaWgaaWcbaGaem4AaSgabeaakiabcIcaOiabdEgaNnaaBaaaleaacqWGPbqAaeqaaOGaeiilaWIaem4zaC2aaSbaaSqaaiabdQgaQbqabaGccqGGPaqkcqGHsislcqWGKbazdaWgaaWcbaGaemiBaWgabeaakmaabmaabaGaem4zaC2aaSbaaSqaaiabdMgaPbqabaGccqGGSaalcqWGNbWzdaWgaaWcbaGaemOAaOgabeaaaOGaayjkaiaawMcaaaGaay5waiaaw2faaaWcbaGaem4zaC2aaSbaaWqaaiabdMgaPbqabaWccqGGSaalcqWGNbWzdaWgaaadbaGaemOAaOgabeaaliabgIGiolabdoeadnaaBaaameaacqWGRbWAcqWGSbaBaeqaaSGaei4oaSJaemyAaKMaeyipaWJaemOAaOgabeqdcqGHris5aaGcbaWaaqWaaeaacqWGdbWqdaWgaaWcbaGaem4AaSMaemiBaWgabeaaaOGaay5bSlaawIa7aaaacqGHRaWkcqWGWbaCdaqadaqaamaaemaabaGaemiwaG1aaSbaaSqaaiabdUgaRbqabaGccqWIQisvcqWGybawdaWgaaWcbaGaemiBaWgabeaaaOGaay5bSlaawIa7aiabgkHiTmaaemaabaGaem4qam0aaSbaaSqaaiabdUgaRjabdYgaSbqabaaakiaawEa7caGLiWoaaiaawIcacaGLPaaacaWLjaGaaCzcamaabmaabaGaeGymaedacaGLOaGaayzkaaaaaa@8F3A@

where *d*_*k*_(*g*_*i*_, *g*_*j*_) = location of *g*_*j *_- location of *g*_*i *_in genome *k*, |*s*| denotes the size of a set *s *and *p *is a parameter to penalize the genomes not sharing the same set of genes. The summation term dictates the conservation of gene order as well as the conservation of gene-to-gene distances between the two genomes. The second term dictates gene co-occurrence.

We represent each genome *k *by a vector of distance values: *F*_*k *_= [*D*_*k*1_, *D*_*k*2_,...,*D*_*km*_] and then we perform K-Means clustering over the set *S *= {*F*_*k *_| *k *= 1,..., *m*}. We implemented an adaptive technique such that the number of clusters grows incrementally until the size of the largest cluster is smaller than a specified threshold. The threshold *t *∈ (0,1] describes the fractional size of the Euclidean space spanned by *S*. Each resulting cluster contains genomes with high resemblance in gene distribution. Alternative adaptive clustering methods include dynamic self-organizing maps [26,27].

### Support Vector Machines for function prediction

**Figure 2:**
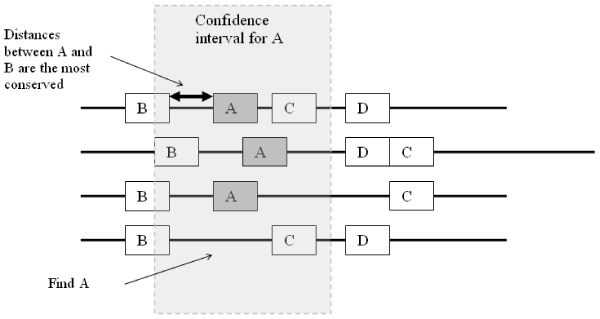
**An illustration of a cluster containing four genomes**. Performing function prediction over gene class "A" consists of two steps: i) perform Leave-One-Out cross validation over the first three genomes and hence adapt to the optimal kernel parameters, ii) find A in the bottom genome within the confidence interval. Since the distances between A and B genes are the most conserved, class B will act as the reference genes for computing relative positions for class A genes for use as one of the training features.

**Figure 3:**
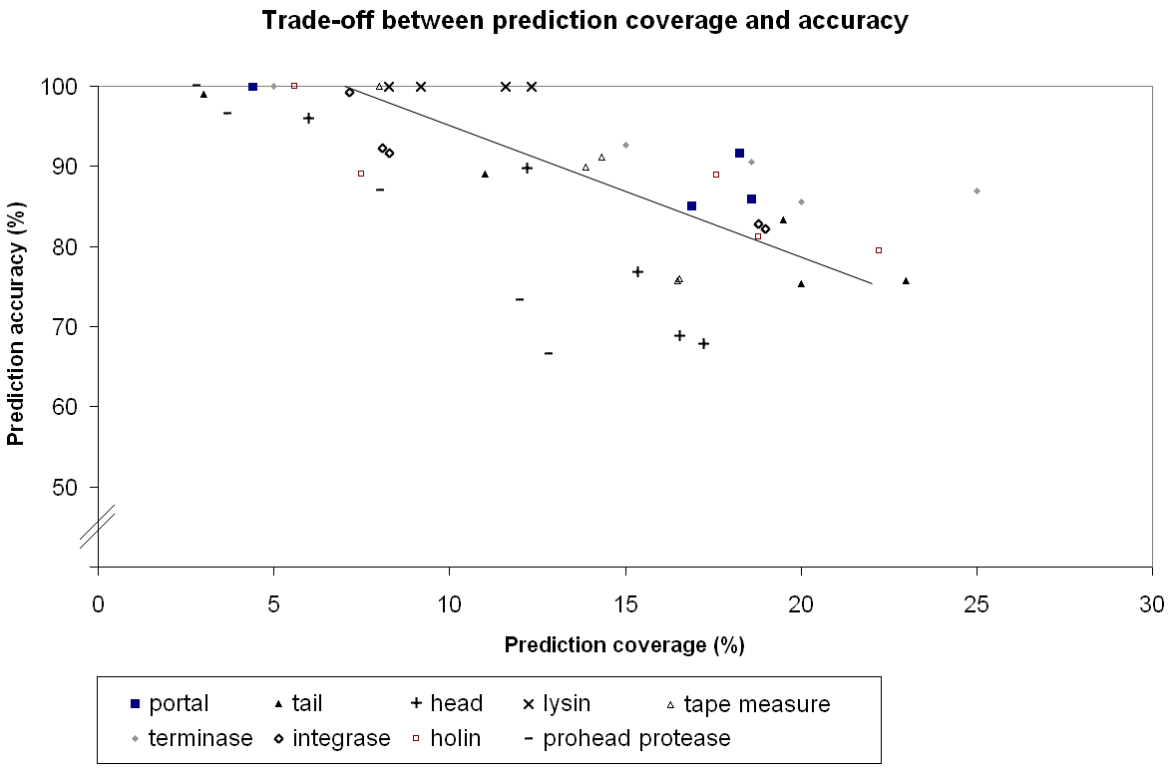
**A plot of cross-validated prediction accuracy versus prediction coverage of the genomes in the database (296)**. Prediction coverage indicates the percentage amount of genomes that have been included to perform the leave-one-out cross validations using SynFPS. The maximum coverage of each gene function is limited by the number of its existences detected in the database. The coverage is varied using different adaptive threshold for the K-Means clustering.

The clusters of genomes are analysed separately and individually in the last stage of the system. For each cluster, we use the information of the previously identified genes to predict the functions of other genes that exhibit similar context. This is achieved by extracting a set of genes from the cluster and converting them into positive and negative training data for a discriminative classification. Positive data are formed by the group of genes previously identified by the system during the match of regular expression plus any manually added genes, with each gene function representing one class. Negative data comprise the genes that are neighbours to the positive genes. The size of neighbourhood is determined by the statistics of the gene locations in that particular cluster. We use 99% confidence interval on the gene locations of each class to determine the range in which neighbour genes are to be included. This interval also determines the set of candidate genes on which function predictions are performed (see Figure 2). The discriminative classification is carried out by a Support Vector Machine (SVM) [28], which has been reported with superior results in a variety of biological applications [29-31]. For each gene function, the SVM produces a binary result on each candidate gene indicating whether or not the gene belongs to that function class. Since the number of gene functions is specified by the user and is not likely to cover every possible function, only a subset of the candidate genes – those with positive results – will eventually be assigned with predicted functions.

To enhance prediction accuracy, we force a unique positive prediction in every genome within a cluster. This is based on an assumption that all pairs of genomes within a cluster would have a one-to-one mapping of genes (gene correspondence). The decision values generated by SVM depict the relative positiveness of each candidate gene. Consequently, the gene with the strongest decision value will be chosen as the positive prediction.

In order to apply SVM, each gene is converted into a numeric vector capturing the following features: composition, normalized van der Waals volume, hydrophobicity, polarity [30,32], pairwise similarity scores against other genes in the database [29], relative position and gene size. To compute the "relative position", the system first finds the gene class which has the most conserved distance to the gene under current prediction. For example, as demonstrated in Figure 2, if we are making predictions over class A, then class B will be chosen as the reference for computing the relative positions because the distances between class B genes and class A genes are the most conserved. The relative position of a gene in class A is then computed as the distance between itself and the class B gene in the corresponding genome.

The pairwise similarity scores have been observed to improve classification accuracies. These scores represent the distance between a gene and every other gene in the database [29]. However, it should be emphasized that while these sequence similarity scores enhance the strength of the feature vectors, the system does not rely upon similarity significances to detect gene correspondence.

## Availability and requirements

**Project name**: SynFPS

**Project website**: 

**Operating system**: Microsoft Windows family

**Other requirements**: Microsoft .NET Framework 2.0 (free), Bioperl 1.4 (optional)

**Any restrictions to use by non-academics**: None

## Abbreviations

**CDS **Coding Sequence; **HGT **Horizontal gene transfers; **LOO **Leave-one-out; **SVM **Support Vector Machines; **SynFPS **Synteny-based Function Prediction System

## Competing interests

The authors declare that they have no competing interests.

## Authors' contributions

JL conceived of the study, designed the software and drafted the manuscript. SKH supervised the work and participated in results evaluation. ST conceived of the clustering design and gave expertise in bacteriophage analysis. CIK participated in the SVM predictions. All authors have participated in preparing the manuscript, have read and approved the final manuscript.

## Supplementary Material

Additional file 1 Supplementary material – the list of all phages and clustering result Click here for file

## Figures and Tables

**Table 1:** Regular expression patterns used for the nine selected genes.

Gene	Search pattern
Major head	(?<!minor)\b(head|capsid)\b
Major tail	(?<!minor)\btail\b
Terminase (large subunit)	terminase|\bterL\b
Holin	\bholin\b
Lysin	\blysin\b
Tape measure	\btape\b|minor tail
Integrase	integrase
Portal protein	\bportal\b
Prohead protease	prohead AND protease^†^

† Not a direct regular expression; "Prohead" and "protease" were searched separately and the results were combined using the AND operation provided by SynFPS.

These patterns were matched against the CDS annotations of the phages retrieved from GenBank. Note that the search results were then refined via manual inspection. \w – alphanumeric character; \b – word boundary; | – 'or'; * – zero or more of the preceding character.

**Table 2:** Percentage of genes detected using sequence alignment.

Reference Genome		Terminase		Portal		Head		Tail		Tape measure		Prohead protease		Lysin		Holin		Integrase	
		
	E-value cutoff	0.01	0.1	0.01	0.1	0.01	0.1	0.01	0.1	0.01	0.1	0.01	0.1	0.01	0.1	0.01	0.1	0.01	0.1
Bacteriophage bIL285		**31**	37	33	50	-	-	4	19	-	-	46	49	16	18	11	23	57	64
*Lactococcus *phage TP901-1		8	22	13	19	12	**27**	7	22	**96**	**98**	-	-	30	48	13	13	3	15
*Enterobacteria *phage HK97		29	40	35	43	4	26	**12**	19	83	96	**54**	**64**	0	4	5	24	54	**73**
Bacteriophage phi LC3		19	**42**	9	25	**14**	24	4	**25**	63	83	-	-	**36**	**48**	13	14	58	65
*Staphylococcus aureus *phage phi 13		12	25	**40**	**56**	9	19	-	-	77	94	36	38	-	-	**13**	**26**	**62**	71

The percentages are calculated by dividing the number of significantly similar sequences by the total number of sequences found by using regular expression. Sequence similarity is determined by BLAST (bl2seq) [33] using BLOSUM45 with indicated E-value cutoffs. Each sequence is "blasted" against its corresponding gene in the reference genome. The best cases are highlighted in bold.

**Table 3:** Prediction settings and results for the nine gene functions.

	Terminase	Portal	Head	Tail	Tape measure	Prohead protease	Lysin	Holin	Integrase
# positive samples	93	83	26	26	21	11	25	69	67
# negative samples	308	195	107	133	82	28	45	213	102
# clusters	17	15	7	6	7	4	6	16	12
**Prediction Accuracy at *t *= 0.1(%)**	**86.9**	**85.89**	**67.87**	**83.33**	**75.68**	**66.67**	**100**	**79.5**	**82.18**

The total number (#) of positive training samples, negative training samples and the number of clusters involved with each gene class are shown. Accuracy values are computed using leave-one-out cross validations. K-Means adaptive threshold *t *= 0.1. GRBF kernel's *σ *= 2 for Head and Tail; *σ *= 11.3 for all other cases.

**Table 4:** Gene function prediction results for bacteriophage genomes.

**Gene (Phage abbrev.^†^: CDS location)**	**Existing function annotation**	**Predicted function**	**Supporting phages (phage abbrev.^†^)**	**SS**
69: 4704..5324	*Uncharacterised*	Prohead protease	PVL	N
phi-105: 7918..8520	*Uncharacterised*	Major tail protein	Cherry, Gamma, 3A, 47	Y
Tuc2009: 23727..24224	*Uncharacterised*	Major tail protein	bIL285, bIL286, bIL309, ul36, phiSLT	Y
A118: 4590..5159	determines size and shape of viral capsid, putative scaffolding protein	Prohead protease	PVL	N
71: 4149..4748	Phage minor structural protein, GP20	Prohead protease	PVL	N
phi ETA: 21172..21768	minor capsid protein	Prohead protease	phi 13	N
phi 11: 21115..21750	phi Mu50B-like protein	Prohead protease	phi 13	N
P22: 38551..38991	lysozyme, endolysin_autolysin	Lysin	V	N
Sf6: 3975..4859	putative scaffolding protein	Prohead protease	ST64B, V	N
HK620: 23655..24539	scaffold protein	Prohead protease	P27	N
sk1: 8582..11581	Mu-like prophage protein, phage-related protein [function unknown]	Tape measure	bIL170	Y
77: 19572..21026	CHAP domain, Ami_3, SH3 domain	Lysin	phi-105	N
77: 3291..4028	Clp protease	Prohead protease	Cherry, Gamma, phi-105	N
phiSLT: 20002..20775	protease, clp protease	Prohead protease	bIL285, bIL309, phiPV83	N
phiSLT: 38923..40377	amidase, CHAP, Ami_3, SH3b	Lysin	bIL285, bIL286, bIL309, ul36, 315.5, 315.6	Y
bIL286: 21258..21965	protease, clp protease	Prohead protease	bIL285, bIL309, phiPV83	N

**† **Full names of the phages are as follows with abbreviations in bold: *Staphylococcus aureus *bacteriophage **PVL**, Bacteriophage **69**, Bacteriophage **A118**, Bacteriophage **71**, Bacteriophage **phi ETA**, *Staphylococcus aureus *phage **phi 11**, *Staphylococcus aureus *phage **phi 13**, Enterobacteria phage **P22**, Enterobacteria phage **Sf6**, *Salmonella typhimurium *bacteriophage **ST64B**, *Shigella flexneri *bacteriophage **V**, Bacteriophage **HK620**, Bacteriophage **P27**, Bacteriophage **sk1**, Bacteriophage **bIL170**, *Bacillus anthracis *phage **Cherry**, *Bacillus anthracis *phage **Gamma**, Bacteriophage **3A**, Bacteriophage **47**, Bacteriophage **phi-105**, Bacteriophage **77**, Bacteriophage **bIL285**, Bacteriophage **bIL286**, Bacteriophage **bIL309**, Bacteriophage **Tuc2009**, *Lactococcus *phage **ul36**, *Staphylococcus aureus *prophage **phiPV83**, *Staphylococcus aureus *temperate phage **phiSLT**, *Streptococcus pyogenes *phage **315.5**, *Streptococcus pyogenes *phage **315.6**.

This is a subset of the predictions generated by SynFPS. SS refers to Sequence Similarity: N indicates there is no significance in sequence similarity between the target gene (first column) and any of the corresponding genes in the supporting phages (second last column) within the same cluster; Y indicates at least one of the corresponding genes show significant similarity. BLAST-P with Blosum45 has been used to test for similarity significance.
