# Supplementary material for: Gene function prediction based on genomic context clustering and discriminative learning: an application to bacteriophages
Source: BMC Bioinformatics. 2007 May 22;8(Suppl 4):S6. doi: 10.1186/1471-2105-8-S4-S6 (PMC1892085; doi:10.1186/1471-2105-8-S4-S6)
Supplement: Additional file 1 — Supplementary material – the list of all phages and clustering result [file 1471-2105-8-S4-S6-S1.doc]

# Gene function prediction based on genomic context clustering and discriminative learning: an application to bacteriophages

Jason Li^1^, Saman K. Halgamuge^1^, Christopher I. Kells^1^, Sen-Lin Tang^2§^

^1^Dynamic Systems & Control Group, DoMME, University of Melbourne, Melbourne, Australia

^2^Research Center for Biodiversity, Academia Sinica, Taipei, Taiwan

^§^Corresponding author

Email addresses:

JL: [lij@mame.mu.oz.au](mailto:lij@mame.mu.oz.au)

SKH: saman@unimelb.edu.au

CIK: c.kells@ugrad.unimelb.edu.au

ST: sltang@gate.sinica.edu.tw

# Supplementary Material

**LIST OF TABLES**

[**Table 1. List of all completely sequenced phages used.** Annotation files were retrieved from NCBI GenBank on 17 Oct, 2005. The phages are listed in two groups according to whether or not their genomes contain at least one of the inspected genes: major head, major tail, terminase, portal protein, prohead protease, tape measure, integrase, holin and lysin. 2](#__RefHeading___Toc147638570)

**LIST OF FIGURES**

[Figure 1. Genome clustering result for K-Means adaptive threshold t=0.1. This figure contains only clusters that have been used in the function prediction stage; junk clusters are not included. 9](#__RefHeading___Toc153779865)

Table 1. List of all completely sequenced phages used. Annotation files were retrieved from NCBI GenBank on 17 Oct, 2005. The phages are listed in two groups according to whether or not their genomes contain at least one of the inspected genes: major head, major tail, terminase, portal protein, prohead protease, tape measure, integrase, holin and lysin.

| **Phage** | **Class** | **Size (bp)** | **Accession #** |
| --- | --- | --- | --- |
| ***Genomes containing at least one of the inspected genes*** | | | |
| Acholeplasma phage L2 | Plasmavirus | 11965 | NC_001447 |
| Actinoplanes phage phiAsp2 | Viruses | 58638 | NC_005885 |
| Acyrthosiphon pisum bacteriophage APSE-1 | Podoviridae | 36524 | NC_000935 |
| Aeromonas phage 31 | Myoviridae | 172963 | NC_007022 |
| Bacillus anthracis phage Cherry | Siphoviridae | 36615 | NC_007457 |
| Bacillus anthracis phage Gamma | Siphoviridae | 37253 | NC_007458 |
| Bacillus clarkii bacteriophage BCJA1c | Siphoviridae | 41092 | NC_006557 |
| Bacillus phage GA-1 (GA-1) | phi-29-like viruses | 21129 | NC_002649 |
| Bacillus phage phi29 (Bacillus phage PZA) | phi-29-like viruses | 19366 | NC_001423 |
| Bacillus thuringiensis bacteriophage Bam35c | Tectivirus | 14935 | NC_005258 |
| Bacillus thuringiensis phage GIL16c | unclassified Tectivirus | 14844 | NC_006945 |
| Bacteriophage 11b | Viruses | 36107 | NC_006356 |
| Bacteriophage 187 | Siphoviridae | 39620 | NC_007047 |
| Bacteriophage 2638A | Siphoviridae | 41318 | NC_007051 |
| Bacteriophage 29 | Siphoviridae | 42802 | NC_007061 |
| Bacteriophage 37 | Siphoviridae | 43681 | NC_007055 |
| Bacteriophage 3A | Siphoviridae | 43095 | NC_007053 |
| Bacteriophage 42e | Siphoviridae | 45861 | NC_007052 |
| Bacteriophage 44RR2.8t | T4-like viruses | 173591 | NC_005135 |
| Bacteriophage 47 | Siphoviridae | 44777 | NC_007054 |
| Bacteriophage 52A | Siphoviridae | 41690 | NC_007062 |
| Bacteriophage 53 | Siphoviridae | 43883 | NC_007049 |
| Bacteriophage 55 | Siphoviridae | 41902 | NC_007060 |
| Bacteriophage 66 | Podoviridae | 18199 | NC_007046 |
| Bacteriophage 69 | Siphoviridae | 42732 | NC_007048 |
| Bacteriophage 71 | Siphoviridae | 43114 | NC_007059 |
| Bacteriophage 77 | Siphoviridae | 41708 | NC_005356 |
| Bacteriophage 85 | Siphoviridae | 44283 | NC_007050 |
| Bacteriophage 88 | Siphoviridae | 43231 | NC_007063 |
| Bacteriophage 92 | Siphoviridae | 42431 | NC_007064 |
| Bacteriophage 933W | Lambda-like viruses | 61670 | NC_000924 |
| Bacteriophage 96 | Siphoviridae | 43576 | NC_007057 |
| Bacteriophage A118 | Siphoviridae | 40834 | NC_003216 |
| Bacteriophage Aaphi23 | Myoviridae | 43033 | NC_004827 |
| Bacteriophage Aeh1 (Aeh1) | T4-like viruses | 233234 | NC_005260 |
| Bacteriophage B103 | phi-29-like viruses | 18630 | NC_004165 |
| Bacteriophage B3 | Siphoviridae | 38439 | NC_006548 |
| Bacteriophage bIL170 | Siphoviridae | 31754 | NC_001909 |
| Bacteriophage bIL285 | Siphoviridae | 35538 | NC_002666 |
| Bacteriophage bIL286 | Siphoviridae | 41834 | NC_002667 |
| Bacteriophage bIL309 | Siphoviridae | 36949 | NC_002668 |
| Bacteriophage bIL310 | Siphoviridae | 14957 | NC_002669 |

| Table 1 *(continued)* | | | |
| --- | --- | --- | --- |
| **Phage** | **Class** | **Size (bp)** | **Accession #** |
| Bacteriophage bIL311 | Siphoviridae | 14510 | NC_002670 |
| Bacteriophage bIL312 | Siphoviridae | 15179 | NC_002671 |
| Bacteriophage D3112 | Lambda-like viruses | 37611 | NC_005178 |
| Bacteriophage EJ-1 | Myoviridae | 42935 | NC_005294 |
| Bacteriophage EW | Siphoviridae | 45286 | NC_007056 |
| Bacteriophage Felix 01 | Caudovirales | 86155 | NC_005282 |
| Bacteriophage G1 | Myoviridae | 138715 | NC_007066 |
| Bacteriophage HK620 | Siphoviridae | 38297 | NC_002730 |
| Bacteriophage JK06 | Siphoviridae | 46072 | NC_007291 |
| Bacteriophage K139 | K139-like phages | 33106 | NC_003313 |
| Bacteriophage KVP40 | T4-like viruses | 244835 | NC_005083 |
| Bacteriophage L-413C | P2-like viruses | 30728 | NC_004745 |
| Bacteriophage L5 | Viruses | 2435 | NC_003695 |
| Bacteriophage lambda | Lambda-like viruses | 48502 | NC_001416 |
| Bacteriophage Mx8 | P22-like viruses | 49534 | NC_003085 |
| Bacteriophage P27 | Lambda-like viruses | 42575 | NC_003356 |
| Bacteriophage P4 | P2-like viruses | 11624 | NC_001609 |
| Bacteriophage phBC6A51 | Viruses | 61395 | NC_004820 |
| Bacteriophage phBC6A52 | Viruses | 38472 | NC_004821 |
| Bacteriophage phi AT3 | Viruses | 39166 | NC_005893 |
| Bacteriophage phi CTX | P2-like viruses | 35580 | NC_003278 |
| Bacteriophage phi ETA | Siphoviridae | 43081 | NC_003288 |
| Bacteriophage phi JL001 | Siphoviridae | 63649 | NC_006938 |
| Bacteriophage phi LC3 | Siphoviridae | 32172 | NC_005822 |
| Bacteriophage phi1026b | Lambda-like viruses | 54865 | NC_005284 |
| Bacteriophage phi-105 | Lambda-like viruses | 39325 | NC_004167 |
| Bacteriophage phi3626 | Siphoviridae | 33507 | NC_003524 |
| Bacteriophage phi-BT1 | PhiC31-like viruses | 41831 | NC_004664 |
| Bacteriophage phi-C31 | PhiC31-like viruses | 41491 | NC_001978 |
| Bacteriophage phiE125 | Lambda-like viruses | 53373 | NC_003309 |
| Bacteriophage phig1e | Siphoviridae | 42259 | NC_004305 |
| bacteriophage phiKMV | T7-like viruses | 42519 | NC_005045 |
| Bacteriophage phiKO2 | Siphoviridae | 51601 | NC_005857 |
| Bacteriophage phiMFV1 | Caudovirales | 15141 | NC_005964 |
| Bacteriophage phiYeO3-12 | T7-like viruses | 39600 | NC_001271 |
| Bacteriophage PSP3 | P2-like viruses | 30636 | NC_005340 |
| Bacteriophage PT1028 | Viruses | 15603 | NC_007045 |
| Bacteriophage PY54 | Siphoviridae | 46339 | NC_005069 |
| Bacteriophage r1t | Siphoviridae | 33350 | NC_004302 |
| Bacteriophage RM 378 | T4-like viruses | 129908 | NC_004735 |
| Bacteriophage ROSA | Viruses | 43155 | NC_007058 |
| Bacteriophage sk1 | Siphoviridae | 28451 | NC_001835 |
| Bacteriophage SPBc2 | Siphoviridae | 134416 | NC_001884 |
| Bacteriophage S-PM2 | Myoviridae | 196280 | NC_006820 |
| Bacteriophage SPP1 | Lambda-like viruses | 44007 | NC_004166 |
| Bacteriophage Tuc2009 | Siphoviridae | 38347 | NC_002703 |

| Table 1 *(continued)* | | | |
| --- | --- | --- | --- |
| **Phage** | **Class** | **Size (bp)** | **Accession #** |
| Bacteriophage VT2-Sa | Lambda-like viruses | 60942 | NC_000902 |
| Bacteriophage VWB | Lambda-like viruses | 49220 | NC_005345 |
| Bacteriophage WPhi | P2-like viruses | 32684 | NC_005056 |
| Bacteriophage X2 | Siphoviridae | 43440 | NC_007065 |
| Bordetella phage BIP-1 | Podoviridae | 42638 | NC_005809 |
| Bordetella phage BMP-1 | Podoviridae | 42663 | NC_005808 |
| Bordetella phage BPP-1 | Podoviridae | 42493 | NC_005357 |
| Burkholderia cenocepacia phage Bcep1 | Myoviridae | 48177 | NC_005263 |
| Burkholderia cenocepacia phage BcepB1A | Myoviridae | 47399 | NC_005886 |
| Burkholderia cenocepacia phage BcepMu | Mu-like viruses | 36748 | NC_005882 |
| Burkholderia cepacia complex phage BcepC6B | Caudovirales | 42415 | NC_005887 |
| Burkholderia cepacia phage Bcep22 | Podoviridae | 63882 | NC_005262 |
| Burkholderia cepacia phage Bcep43 | Myoviridae | 48024 | NC_005342 |
| Burkholderia cepacia phage Bcep781 | Myoviridae | 48247 | NC_004333 |
| Burkholderia cepacia phage BcepNazgul | Caudovirales | 58128 | NC_005091 |
| Burkholderia pseudomallei phage phi52237 | Viruses | 37639 | NC_007145 |
| Coliphage K1F | Viruses | 39704 | NC_007456 |
| Cyanophage P60 | Podoviridae | 47872 | NC_003390 |
| Cyanophage P-SSM2 | Myoviridae | 252401 | NC_006883 |
| Cyanophage P-SSM4 | Myoviridae | 178249 | NC_006884 |
| Cyanophage P-SSP7 | Podoviridae | 44970 | NC_006882 |
| Enterobacteria phage 186 | P2-like viruses | 30624 | NC_001317 |
| Enterobacteria phage epsilon15 | P22-like viruses | 39671 | NC_004775 |
| Enterobacteria phage HK022 | Lambda-like viruses | 40751 | NC_002166 |
| Enterobacteria phage HK97 | Lambda-like viruses | 39732 | NC_002167 |
| Enterobacteria phage L17 | Viruses | 14935 | NC_007449 |
| Enterobacteria phage Mu | Mu-like viruses | 36717 | NC_000929 |
| Enterobacteria phage N15 | N15-like viruses | 46375 | NC_001901 |
| Enterobacteria phage P1 | P1-like viruses | 94800 | NC_005856 |
| Enterobacteria phage P2 | P2-like viruses | 33593 | NC_001895 |
| Enterobacteria phage P22 | P22-like viruses | 41724 | NC_002371 |
| Enterobacteria phage PR3 | Viruses | 14937 | NC_007450 |
| Enterobacteria phage PR4 | Viruses | 14954 | NC_007451 |
| Enterobacteria phage PR5 | Viruses | 14939 | NC_007452 |
| Enterobacteria phage PR772 | Viruses | 14942 | NC_007453 |
| Enterobacteria phage RB43 | T4-like viruses | 180500 | NC_007023 |
| Enterobacteria phage RB49 (RB49) | T4-like viruses | 164018 | NC_005066 |
| Enterobacteria phage RB69 (RB69) | T4-like viruses | 167560 | NC_004928 |
| Enterobacteria phage Sf6 | P22-like viruses | 39043 | NC_005344 |
| Enterobacteria phage SP6 | T7-like viruses | 43769 | NC_004831 |
| Enterobacteria phage T1 | T1-like viruses | 48836 | NC_005833 |
| Enterobacteria phage T3 | T7-like viruses | 38208 | NC_003298 |
| Enterobacteria phage T4 (T4) | T4-like viruses | 168903 | NC_000866 |
| Enterobacteria phage T5 | T5-like viruses | 121750 | NC_005859 |
| Enterobacteria phage T7 | T7-like viruses | 39937 | NC_001604 |
| Haemophilus phage HP1 | P2-like viruses | 32355 | NC_001697 |
| Haemophilus phage HP2 | P2-like viruses | 31508 | NC_003315 |

| Table 1 *(continued)* | | | |
| --- | --- | --- | --- |
| **Phage** | **Class** | **Size (bp)** | **Accession #** |
| Halorubrum phage HF2 | Haloviruses | 77670 | NC_003345 |
| Lactobacillus bacteriophage phi adh | Siphoviridae | 43785 | NC_000896 |
| Lactobacillus casei bacteriophage A2 | Siphoviridae | 43411 | NC_004112 |
| Lactobacillus johnsonii prophage Lj928 | Siphoviridae | 38384 | NC_005354 |
| Lactobacillus johnsonii prophage Lj965 | Siphoviridae | 40190 | NC_005355 |
| Lactobacillus plantarum bacteriophage LP65 | SPO1-like viruses | 131522 | NC_006565 |
| Lactobacillus plantarum bacteriophage phiJL-1 | Viruses | 36674 | NC_006936 |
| Lactococcus phage BK5-T | Siphoviridae | 40003 | NC_002796 |
| Lactococcus phage c2 | c2-like viruses | 22172 | NC_001706 |
| Lactococcus phage P335 (P335) | Siphoviridae | 36596 | NC_004746 |
| Lactococcus phage TP901-1 | Siphoviridae | 37667 | NC_002747 |
| Lactococcus phage ul36 | Siphoviridae | 36798 | NC_004066 |
| Listeria phage 2389 | Siphoviridae | 37618 | NC_003291 |
| Listonella pelagia phage phiHSIC | Caudovirales | 37966 | NC_006953 |
| Methanobacterium phage psiM2 | Siphoviridae | 26111 | NC_001902 |
| Methanothermobacter wolfeii prophage psiM100 | Siphoviridae | 28798 | NC_002628 |
| Mycobacteria phage D29 | L5-like viruses | 49136 | NC_001900 |
| Mycobacterium phage Bxb1 | Siphoviridae | 50550 | NC_002656 |
| Mycobacterium phage Bxz1 | Myoviridae | 156102 | NC_004687 |
| Mycobacterium phage Bxz2 | Siphoviridae | 50913 | NC_004682 |
| Mycobacterium phage Che8 | Siphoviridae | 59471 | NC_004680 |
| Mycobacterium phage Che9c | Siphoviridae | 57050 | NC_004683 |
| Mycobacterium phage Cjw1 | Siphoviridae | 75931 | NC_004681 |
| Mycobacterium phage Corndog | Siphoviridae | 69777 | NC_004685 |
| Mycobacterium phage L5 | L5-like viruses | 52297 | NC_001335 |
| Mycobacterium phage Omega | Siphoviridae | 110865 | NC_004688 |
| Mycobacterium phage TM4 | Siphoviridae | 52797 | NC_003387 |
| Mycoplasma arthritidis bacteriophage MAV1 | Caudovirales | 15644 | NC_001942 |
| Mycoplasma virus P1 | Podoviridae | 11660 | NC_002515 |
| Phage BP-4795 | Lambda-like viruses | 57930 | NC_004813 |
| Pseudomonas aeruginosa bacteriophage PaP2 | Podoviridae | 43783 | NC_005884 |
| Pseudomonas aeruginosa phage PaP3 | Podoviridae | 45503 | NC_004466 |
| Pseudomonas phage D3 | Siphoviridae | 56425 | NC_002484 |
| Pseudomonas phage F116 | Podoviridae | 65195 | NC_006552 |
| Pseudomonas phage gh-1 (gh-1) | T7-like viruses | 37359 | NC_004665 |
| Pseudomonas phage Pf1 | Inovirus | 7349 | NC_001331 |
| Pseudomonas phage phiKZ | Myoviridae | 280334 | NC_004629 |
| Salmonella typhimurium bacteriophage ES18 | Podoviridae | 46900 | NC_006949 |
| Salmonella typhimurium bacteriophage ST104 | Podoviridae | 41391 | NC_005841 |
| Salmonella typhimurium bacteriophage ST64T | Podoviridae | 40679 | NC_004348 |
| Salmonella typhimurium phage ST64B | Podoviridae | 40149 | NC_004313 |
| Shigella flexneri bacteriophage V | Podoviridae | 37074 | NC_003444 |
| Sinorhizobium meliloti phage PBC5 | Caudovirales | 57416 | NC_003324 |
| Staphylococcus aureus bacteriophage PVL | Siphoviridae | 41401 | NC_002321 |
| Staphylococcus aureus phage phi 11 | Siphoviridae | 43604 | NC_004615 |
| Staphylococcus aureus phage phi 12 | Siphoviridae | 44970 | NC_004616 |
| Staphylococcus aureus phage phi 13 | Siphoviridae | 42722 | NC_004617 |

| *Table 1 (continued)* | | | |
| --- | --- | --- | --- |
| **Phage** | **Class** | **Size (bp)** | **Accession #** |
| Staphylococcus aureus phage phiP68 | phi-29-like viruses | 18227 | NC_004679 |
| Staphylococcus aureus prophage phiPV83 | Siphoviridae | 45636 | NC_002486 |
| Staphylococcus aureus temperate phage phiSLT | Siphoviridae | 42942 | NC_002661 |
| Staphylococcus phage 44AHJD (44AHJD) | phi-29-like viruses | 16784 | NC_004678 |
| Staphylococcus phage K | Myoviridae | 127395 | NC_005880 |
| Staphylococcus phage phiN315 | Siphoviridae | 44082 | NC_004740 |
| Staphylococcus phage Twort | Myoviridae | 130706 | NC_007021 |
| Streptococcus mitis phage SM1 | Siphoviridae | 34692 | NC_004996 |
| Streptococcus phage C1 | phi-29-like viruses | 16687 | NC_004814 |
| Streptococcus phage Cp-1 | phi-29-like viruses | 19343 | NC_001825 |
| Streptococcus pneumoniae bacteriophage MM1 | Siphoviridae | 40248 | NC_003050 |
| Streptococcus pyogenes phage 315.1 | Viruses | 39538 | NC_004584 |
| Streptococcus pyogenes phage 315.2 | Viruses | 41072 | NC_004585 |
| Streptococcus pyogenes phage 315.3 | Viruses | 34419 | NC_004586 |
| Streptococcus pyogenes phage 315.4 | Viruses | 41796 | NC_004587 |
| Streptococcus pyogenes phage 315.5 | Viruses | 38206 | NC_004588 |
| Streptococcus pyogenes phage 315.6 | Viruses | 40014 | NC_004589 |
| Streptococcus thermophilus bacteriophage 2972 | Siphoviridae | 34704 | NC_007019 |
| Streptococcus thermophilus bacteriophage 7201 | Siphoviridae | 35466 | NC_002185 |
| Streptococcus thermophilus bacteriophage DT1 | Siphoviridae | 34815 | NC_002072 |
| Streptococcus thermophilus bacteriophage Sfi11 | Siphoviridae | 39807 | NC_002214 |
| Streptococcus thermophilus bacteriophage Sfi19 | Siphoviridae | 37370 | NC_000871 |
| Streptococcus thermophilus bacteriophage Sfi21 | Siphoviridae | 40739 | NC_000872 |
| Streptococcus thermophilus temperate bacteriophage O1205 | Siphoviridae | 43075 | NC_004303 |
| Sulfolobus spindle-shaped virus 1 | Fusellovirus | 15465 | NC_001338 |
| Sulfolobus spindle-shaped virus 2 | Fusellovirus | 14796 | NC_005265 |
| Sulfolobus spindle-shaped virus Kamchatka-1 | Fusellovirus | 17385 | NC_005361 |
| Sulfolobus spindle-shaped virus Ragged Hills | Fusellovirus | 16473 | NC_005360 |
| Sulfolobus tengchongensis spindle-shaped virus STSV1 | Fusellovirus | 75294 | NC_006268 |
| Temperate phage phiNIH1.1 | Siphoviridae | 41796 | NC_003157 |
| Vibrio harveyi bacteriophage VHML | Myoviridae | 43198 | NC_004456 |
| Vibrio phage VP5 | Podoviridae | 39786 | NC_005891 |
| Vibrio phage VSK | Inovirus | 6882 | NC_003327 |
| Vibriophage VP2 | Podoviridae | 39853 | NC_005879 |
| Vibriophage VP4 | T7-like viruses | 39503 | NC_007149 |
| Vibriophage VpV262 | T7-like viruses | 46012 | NC_003907 |
| Virus PhiCh1 | Myoviridae | 58498 | NC_004084 |
| Xanthomonas campestris pv. pelargonii phage Xp15 | Caudovirales | 55770 | NC_007024 |
| Xanthomonas oryzae bacteriophage Xp10 | Caudovirales | 44373 | NC_004902 |
| Xanthomonas phage Cf1c | Inovirus | 7308 | NC_001396 |
| Yersinia pestis phage phiA1122 | T7-like viruses | 37555 | NC_004777 |
| ***Genomes that do not contain any of the inspected genes*** | | | |
| Acholeplasma phage MV-L1 | Plectrovirus | 4491 | NC_001341 |
| Acidianus filamentous virus 1 | Gammalipothrixvirus | 20869 | NC_005830 |
| Acinetobacter phage AP205 | Levivirus | 4268 | NC_002700 |

| Table 1 *(continued)* | | | |
| --- | --- | --- | --- |
| **Phage** | **Class** | **Size (bp)** | **Accession #** |
| Bacteriophage IN93 | Viruses | 19603 | NC_004462 |
| Bacteriophage KS7 | P22-like viruses | 40794 | NC_006940 |
| Bacteriophage phi-12 | Cystovirus | 6751 | NC_004173 |
| Bacteriophage phi-12 | Cystovirus | 2322 | NC_004174 |
| Bacteriophage phi-12 | Cystovirus | 4100 | NC_004175 |
| Bacteriophage phi-8 | Cystovirus | 7051 | NC_003299 |
| Bacteriophage phi-8 | Cystovirus | 4741 | NC_003300 |
| Bacteriophage phi-8 | Cystovirus | 3192 | NC_003301 |
| Bacteriophage SH1 | Viruses | 30889 | NC_007217 |
| Bacteriophage Vf12 | Inovirus | 7965 | NC_005949 |
| Bacteriophage Vf33 | Inovirus | 7965 | NC_005948 |
| Bacteriophage VfO3K6 | Inovirus | 8784 | NC_002362 |
| Bacteriophage VfO4K68 | Inovirus | 6891 | NC_002363 |
| Bacteriophage VSKK | Inovirus | 6834 | NC_003311 |
| Chlamydia phage 1 | Chlamydiamicrovirus | 4877 | NC_001741 |
| Chlamydia phage 2 | Chlamydiamicrovirus | 4563 | NC_002194 |
| Chlamydia phage 3 | Chlamydiamicrovirus | 4554 | NC_008355 |
| Chlamydia phage 4 | Chlamydiamicrovirus | 4530 | NC_007461 |
| Chlamydia pneumoniae phage CPAR39 | Chlamydiamicrovirus | 4532 | NC_002180 |
| Coliphage ID11 | Microvirus | 5577 | NC_006954 |
| Coliphage phiK | Microvirus | 6089 | NC_001730 |
| Coliphage phiX174 | Microvirus | 5386 | NC_001422 |
| Enterobacteria phage alpha3 | Microvirus | 6087 | NC_001330 |
| Enterobacteria phage FI | Allolevivirus | 4276 | NC_004301 |
| Enterobacteria phage fr | Levivirus | 3575 | NC_001333 |
| Enterobacteria phage G4 | Microvirus | 5577 | NC_001420 |
| Enterobacteria phage GA | Levivirus | 3466 | NC_001426 |
| Enterobacteria phage I2-2 | Inovirus | 6744 | NC_001332 |
| Enterobacteria phage If1 | Inovirus | 8454 | NC_001954 |
| Enterobacteria phage Ike | Inovirus | 6883 | NC_002014 |
| Enterobacteria phage KU1 | Levivirus | 3486 | NC_002250 |
| Enterobacteria phage M13 | Inovirus | 6407 | NC_003287 |
| Enterobacteria phage PRD1 | Tectivirus | 14925 | NC_001421 |
| Enterobacteria phage S13 | Microvirus | 5386 | NC_001424 |
| Enterobacterio phage MS2 | Levivirus | 3569 | NC_001417 |
| Enterobacteriophage Qbeta | Allolevivirus | 4215 | NC_001890 |
| Guinea pig Chlamydia phage | Chlamydiamicrovirus | 4529 | NC_001998 |
| Mycobacteriophage PG1 | Siphoviridae | 68999 | NC_005259 |
| Mycobacterium phage Barnyard | Siphoviridae | 70797 | NC_004689 |
| Mycobacterium phage Che9d | Siphoviridae | 56276 | NC_004686 |
| Mycobacterium phage Rosebush | Siphoviridae | 67480 | NC_004684 |
| Phage phiMH2K | Microvirus | 4594 | NC_002643 |
| Phage phiSMA9 | Viruses | 6907 | NC_007189 |
| Propionibacterium phage phiB5 | Inovirus | 5804 | NC_003460 |
| Pseudoalteromonas phage PM2 | Corticovirus | 10079 | NC_000867 |
| Pseudomonas bacteriophage phi-13 | Cystovirus | 2981 | NC_004170 |
| Pseudomonas bacteriophage phi-13 | Cystovirus | 4213 | NC_004171 |

| Table 1 *(continued)* | | | |
| --- | --- | --- | --- |
| **Phage** | **Class** | **Size (bp)** | **Accession #** |
| Pseudomonas bacteriophage phi-13 | Cystovirus | 6458 | NC_004172 |
| Pseudomonas phage Pf3 | Inovirus | 5833 | NC_001418 |
| Pseudomonas phage phi-6 | Cystovirus | 2948 | NC_003714 |
| Pseudomonas phage phi-6 | Cystovirus | 6374 | NC_003715 |
| Pseudomonas phage phi-6 | Cystovirus | 4063 | NC_003716 |
| Pseudomonas phage PP7 | Levivirus | 3588 | NC_001628 |
| Ralstonia phage p12J | Viruses | 7130 | NC_005131 |
| Roseophage SIO1 | Podoviridae | 39898 | NC_002519 |
| Spiroplasma phage 1-C74 | Plectrovirus | 7768 | NC_003793 |
| Spiroplasma phage 1-R8A2B | Plectrovirus | 8273 | NC_001365 |
| Spiroplasma phage 4 | Spiromicrovirus | 4421 | NC_003438 |
| Stx1 converting bacteriophage | Lambda-like viruses | 59866 | NC_004913 |
| Stx2 converting bacteriophage I | Lambda-like viruses | 61765 | NC_003525 |
| Stx2 converting bacteriophage II | Lambda-like viruses | 62706 | NC_004914 |
| Sulfolobus islandicus filamentous virus | Betalipothrixvirus | 40047 | NC_003214 |
| Sulfolobus islandicus rod-shaped virus 1 | Rudivirus | 32308 | NC_004087 |
| Sulfolobus islandicus rod-shaped virus 2 | Rudivirus | 35450 | NC_004086 |
| Sulfolobus turreted icosahedral virus | Rudivirus | 17663 | NC_005892 |
| SVTS2 plectrovirus | Plectrovirus | 6825 | NC_001270 |
| Vibrio cholerae O139 fs1 phage | Inovirus | 6340 | NC_004306 |
| Vibrio cholerae phage KSF-1phi | Inovirus | 7107 | NC_006294 |
| Vibrio cholerae phage VGJphi | Inovirus | 7542 | NC_004736 |
| Vibrio phage fs2 | Inovirus | 8651 | NC_001956 |


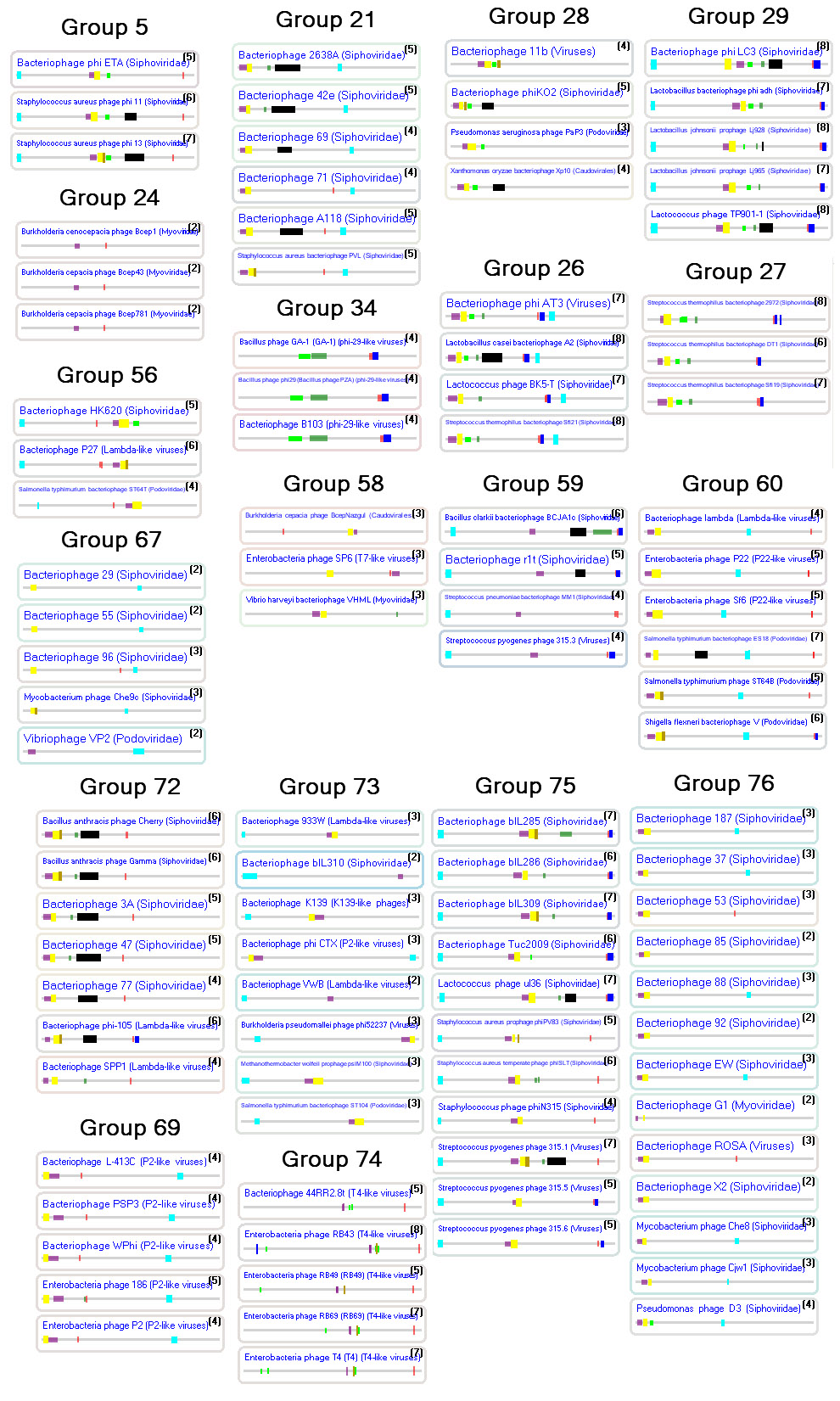


Figure 1. Genome clustering result for K-Means adaptive threshold *t=0.1*. This figure contains only clusters that have been used in the function prediction stage; junk clusters are not included.
